# Posterior root repair with high tibial osteotomy improves radiological and clinical outcomes despite greater medial meniscus extrusion: A comparison of transtibial and all‐inside techniques

**DOI:** 10.1002/jeo2.70956

**Published:** 2026-10-19

**Authors:** Yuki Fujie, Kazunori Yasuda, Koji Yabuuchi, Taku Ebata, Masayuki Inoue, Norimasa Iwasaki, Eiji Kondo

**Affiliations:** ^1^ Department of Orthopaedic Surgery Yagi Orthopaedic Hospital Sapporo Japan; ^2^ Department of Orthopaedic Surgery, Faculty of Medicine and Graduate School of Medicine Hokkaido University Sapporo Japan; ^3^ Centre for Sports Medicine Hokkaido University Hospital Sapporo Japan

**Keywords:** high tibial osteotomy, knee, medial meniscus, meniscus extrusion, meniscus repair, posterior root tear

## Abstract

**Purpose:**

To evaluate whether the transtibial pullout (TTP) or all‐inside suture (AIS) technique provides superior radiological and clinical outcomes in patients undergoing medial meniscus posterior root (MMPR) repair combined with high tibial osteotomy (HTO) over a 24‐ to 48‐month follow‐up.

**Methods:**

This retrospective cohort study analysed 54 knees in 52 patients who underwent MMPR repair with HTO between April 2018 and March 2022. The TTP technique was used until March 2020, after which the AIS technique was performed thereafter. Inclusion criteria were MMPR repair with HTO within 3 months of symptom onset, age 50–70 years, and ≥2 year postoperative follow‐up. Exclusion criteria were non‐index limb symptoms and unrelated postoperative conditions. Ultimately, 28 knees were assigned to the TTP group and 26 to the AIS group. Radiological, arthroscopic and clinical outcomes were evaluated at a uniform follow‐up interval (TTP: 37.1 ± 8.6 months; AIS: 33.8 ± 9.1 months). Medial meniscus extrusion (MME) was quantified using the modified Costa method. Appropriate statistical tests were applied for intra‐ and inter‐group comparisons.

**Results:**

The mean ages were 61.7 ± 7.6 years (TTP) and 62.3 ± 8.5 years (AIS). Baseline demographics, osteoarthritis (OA) grades and HTO types showed no significant differences. Postoperative OA grades did not progress significantly. Repair site continuity was restored in 89.3% of the TTP group and 88.5% of the AIS group. MME ratio increased significantly in both groups (TTP: 49.6% → 62.0%, *p* = 0.003; AIS: 48.8% → 68.8%, *p* < 0.001), with no inter‐group difference. No complications occurred in each group. Lysholm scores improved to 96.3 ± 4.5 (TTP) and 92.8 ± 8.1 (AIS), and all Knee Injury and Osteoarthritis Outcome Score subscales improved significantly in both groups, again without inter‐group differences.

**Conclusions:**

In this patient population, the TTP or AIS techniques performed in conjunction with HTO comparably improved radiological and clinical outcomes at 2–4 years of follow‐up despite increased MME.

**Level of Evidence:**

Level III prospective cohort study.

**Abbreviations**
AIS: all‐inside suture
CI: confidence interval
FTA: lateral femorotibial angle
HKA: hip–knee–ankle angle
HTO: high tibial osteotomy
iV: inverted V‐shaped
IW: inner width
K–L: Kellgren–Lawrence
KOOS: Knee Injury and Osteoarthritis Outcome Score
MA: mechanical axis
MM: medial meniscus
MME: medial meniscus extrusion
MOW: medial opening wedge
MPTA: medial proximal tibial angle
MRI: magnetic resonance imaging
MTP: medial tibial plateau
OA: osteoarthritis
PR: posterior root
TTP: transtibial pullout

## INTRODUCTION

The transtibial pullout (TTP) technique and the all‑inside suture (AIS) technique are commonly used surgical options for repairing medial meniscus posterior root (MMPR) tears. Several systematic reviews and meta‐analyses [2, 4, 11] have reported that MMPR repair using the TTP technique provides functional benefits, with consistent improvements in clinical outcome scores compared with conservative treatment or arthroscopic partial meniscectomy. Cohort studies evaluating the AIS technique have likewise demonstrated favourable clinical outcomes compared with partial meniscectomy or conservative treatment [3, 7, 8, 12, 34]. An important clinical question has therefore emerged: Which of the two techniques, TTP or AIS, provides superior clinical outcomes? Several studies have attempted to address this issue [17, 21, 34], yet their findings remain inconsistent. Some have reported better outcomes with the TTP technique [17, 21], whereas others have suggested that AIS offers superior results [34]. Consequently, the relative superiority of these two repair techniques remains undetermined.

Patients with an MMPR tear who have undergone conservative treatment for OA before developing the tear often elect to undergo MMPR repair combined with high tibial osteotomy (HTO) to achieve a reliable return to sports or work. However, only a few studies have investigated the clinical outcomes of MMPR repair performed in conjunction with HTO [15, 20, 24], and these studies have also reported substantial inconsistencies regarding the relative superiority of the two repair techniques. Thus, clinical evidence for MMPR repair combined with HTO remains insufficient, and the comparative effectiveness of the two techniques has yet to be clarified. To address this gap, a retrospective cohort study was conducted involving patients with Kellgren–Lawrence (K–L) [16] Grade 1–3 knee OA who underwent MMPR repair using either the TTP or AIS technique in conjunction with HTO. This severity range was selected because most previous MMPR repair studies have examined cohorts with similar osteoarthritic grades [2, 3, 4, 7, 8, 11, 12, 34]. The purpose of this study was to address the following research question: In patients undergoing MMPR repair combined with HTO, which of the TTP and AIS techniques yields superior radiological and clinical outcomes over a 2‐ to 4‐year follow‐up period?

As noted above, previous studies of MMPRT repair without HTO have not demonstrated clear superiority of either technique, suggesting minimal differences in their effectiveness. Furthermore, in MMPRT repair performed in conjunction with HTO, any small difference between techniques may be further obscured by the unloading effect of HTO on the medial compartment. Accordingly, it was hypothesized that, in this patient population, the two repair techniques would yield comparable radiological and clinical outcomes at 24–48 months of follow‐up.

## METHODS

### Study design

The following protocol of this retrospective cohort study was approved by the ethics review board of Yagi Orthopaedic Hospital (No. H29‐0001). Informed consent for the use of clinical data in research was obtained from all patients.

Among patients who underwent MMPR repair combined with HTO at our hospital between April 2018 and March 2022, 77 knees from 75 patients met all of the following inclusion criteria: (1) patients aged 50–70 years; (2) patients presenting with new‐onset severe posteromedial knee pain despite appropriate conservative treatment; (3) MRI findings clearly demonstrating an MMPR tear; (4) radiographs showing medial knee osteoarthritis (OA) of K–L Grade 1–3 with varus deformity; (5) patients who elected to undergo the combined procedure after receiving a thorough explanation of the advantages and disadvantages of MMPR repair, unicompartmental knee arthroplasty and total knee arthroplasty and (6) patients who underwent the combined surgeries within 3 months of pain onset. The exclusion criteria were as follows: (1) pain or impairment in the contralateral knee or in either hip or ankle joint, (2) patients who developed incidental medical conditions or sustained trauma after surgery and (3) patients for whom postoperative evaluations could not be completed.

This study employed a sequential design. In the first subperiod (April 2018 to March 2020), consecutive 41 knees of 41 patients (6 men and 35 women) underwent the MMPR repair using the TTP technique reported by Woodmass et al. [30] in conjunction with HTO. In the following subperiod (April 2020 to March 2022), the remaining 36 knees of 34 patients (12 men and 22 women) underwent the MMPR repair using the AIS technique reported by Dragoo et al. [7] in conjunction with HTO. In each group, HTO surgeries were performed according to the following guidelines [33]: (1) When the planned tibial correction angle is less than 10°, medial opening wedge (MOW) HTO is performed. (2) When the planned tibial correction angle is 10° or more, inverted V‐shaped (iV) HTO [1, 18] is performed to reduce the incidence of postoperative complications, including lateral hinge fractures.

All operations were performed by a senior knee surgeon (K.Y.) who had sufficient clinical experience of both arthroscopic meniscus repair and HTO. The same rehabilitation protocol was used after surgery for all knees. The authors advised patients who were more than 1 year postoperative to attend an annual follow‐up visit, during which radiological examinations and clinical evaluations were performed.

### Arthroscopic MMPR repair techniques

#### TTP technique

The TTP technique was carried out following the report described by Woodmass et al. [30]. Briefly, a guide wire was inserted from the anteromedial aspect of the tibia to the MMPR footprint using a wire guide. A 4.5‐mm tibial tunnel was then created with a cannulated drill. A retrograde drill (FlipCutter™; Arthrex Inc.) with a 6.0‐mm diameter was inserted through this tunnel, and a 10‐mm‐deep bone socket was created at the MMPR footprint. Number‐2 polyester sutures (ETHIBOND EXCEL™; Ethicon) were used to repair the MMPR tear. An AIS passer (Knee Scorpion™; Arthrex Inc.) was introduced through the medial portal. The first polyester suture was passed through the apparently intact meniscal substance and tied using a sliding‐knot technique. The second suture was placed 3–5 mm from the first in the same manner. The third lead suture was positioned at the meniscal stump. These three sutures were retrieved separately through the tibial tunnel. The meniscal stump was drawn as deeply as possible into the tibial bone socket by pulling the three sutures (Figure 1a). Finally, the sutures were tied over a small metal button on the tibial cortex.

**Figure 1:**
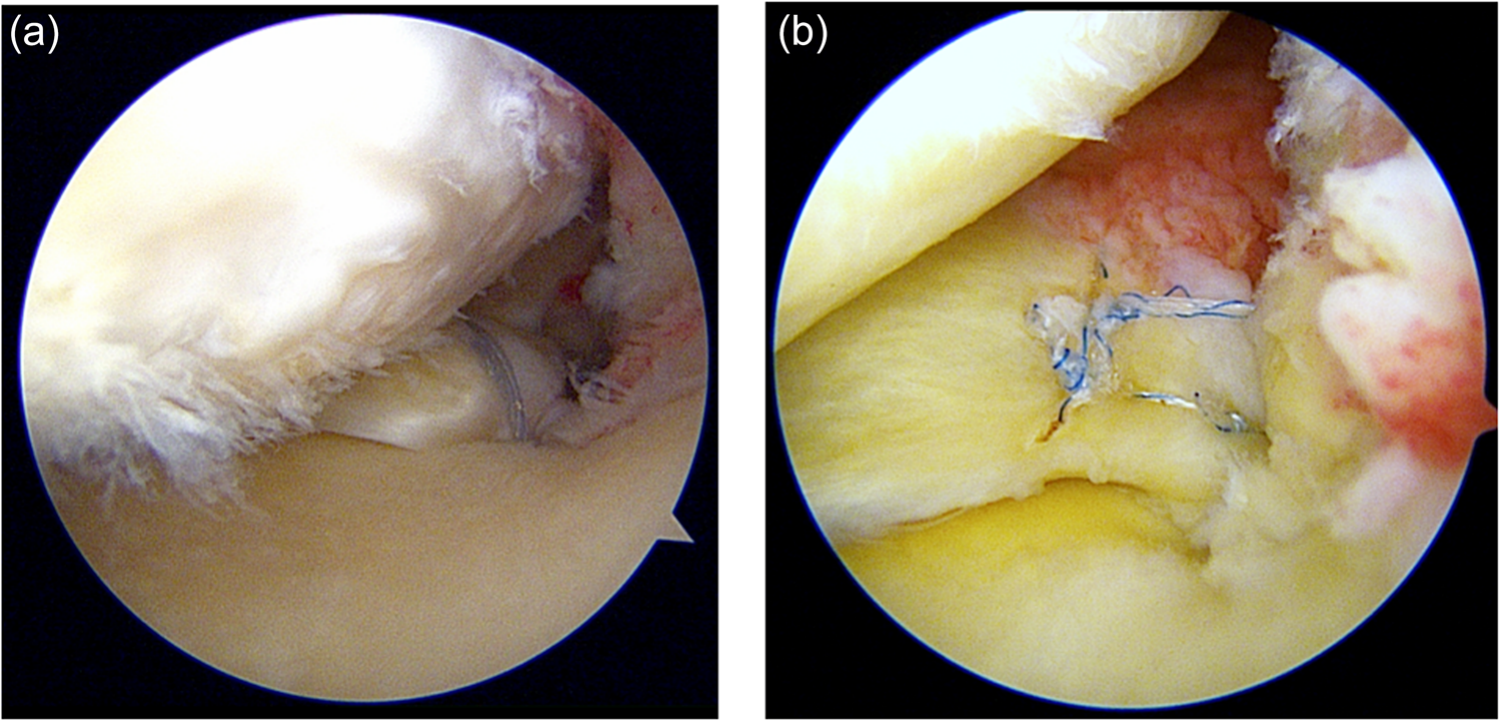
Arthroscopic MMPR repair techniques. (a) Transtibial pull‐out technique. The end of the meniscal stump was drawn as deeply as possible into the tibial bone socket. (b) All‐inside suture technique. The first and second sutures tether the apparently intact meniscal substance to the root remnant on the tibial plateau. The third vertical mattress suture anchors the posterior horn to the posterior capsule. MMPR, medial meniscus posterior root.

#### AIS technique

The AIS technique was carried out following the report described by Dragoo et al. [7]. Briefly, an AIS device (FAST‐FIX 360™; Smith & Nephew Endoscopy) was introduced through the medial portal. The first arm of the device was inserted into the apparently intact meniscal substance at a point 5–8 mm from the edge. The second arm was inserted into the posterior cruciate ligament, penetrating the root remnant on the tibial plateau. The suture was tensioned to reduce the meniscus back to the root remnant. This technique was repeated with a second device placed more anteriorly or posteriorly on the meniscus than the initial suture. Finally, the posterior horn near the suture site was anchored to the posterior knee capsule with a third vertical‐mattress suture to reinforce the repair construct (Figure 1b).

### HTO procedures

#### MOW HTO procedure

This procedure has previously been reported in detail [26, 31]. Briefly, the correction angle was determined preoperatively so that the postoperative %MA would be 65%. The proximal medial tibia was exposed through a 7‐cm medial oblique incision. A pair of Kirschner wires (K‐wires) was inserted into the tibia from a point 40 mm distal to the joint line toward the proximal tibiofibular joint. A biplanar osteotomy was performed along the inserted K‐wires using a thin bone saw and a thin chisel. The osteotomy planes were gradually opened with a spreader until the preoperatively planned correction angle was achieved. Two wedge‐shaped spacers made of beta‐tricalcium phosphate (Osferion 60 Marvelous; Osferion Biomaterials Corp.) were implanted into the opening gap. The osteotomy site was then fixed with a locking plate (TriS^TM^ Medial HTO Plate System; Osferion Biomaterials Corp.) placed on the medial side of the tibia.

#### iV‐shaped HTO (iVHTO) procedure

The details of the iVHTO procedure have been previously reported [13, 18, 28]. In brief, the correction angle was determined preoperatively so that the postoperative %MA would be 65%. Under fluoroscopic guidance, a K‐wire was inserted at the preoperatively planned apex point of the V‐shaped osteotomy, perpendicular to the anterior surface of the proximal tibia. A wire‐insertion guide, set at the preoperatively planned correction angle, was attached to the apex K‐wire, and a pair of guide K‐wires was inserted toward the apex wire through this guide. After a coronal ascending osteotomy was performed, a lateral hemi‐wedge osteotomy was then made along the two K‐wires. A parallel drill guide was mounted on the apex K‐wire, and multiple 2‐mm parallel holes were created in the medial tibia. A medial hemi‐osteotomy was performed along these drill holes. After valgus correction of the tibia, the osteotomy site was fixed with a locking compression plate (TriS^TM^ Lateral HTO Plate System; OsferionBiomaterials Corp.) placed on the lateral side. Finally, morselized bone chips prepared from the resected wedge were implanted into the medial opening gap.

### Postoperative management

All patients underwent postoperative management using the same rehabilitation protocol. The knee was immobilized with a brace, but not tightly, for the first 2 weeks. Straight leg raising and quadriceps‐setting exercises were encouraged on the day after surgery. A range of motion from 0° to 90° of flexion was permitted from Weeks 2–5, and from 0° to 120° from Weeks 5–8. Half weight‐bearing with two crutches was permitted starting 3 weeks after surgery, followed by full weight‐bearing at 5 weeks postoperatively.

### Radiographic evaluations

Anteroposterior (AP) radiographs of the knee and the whole lower limb were taken in the standing position. On an AP radiograph of the knee, the K–L grade for medial OA was determined. On the whole‐lower‐limb radiograph, alignment parameters of the lower limb—including the anatomical femorotibial angle (FTA), anatomical medial proximal tibial angle (MPTA), hip–knee–ankle angle (HKA) and the percentage at which the mechanical axis (MA) (weight‐bearing line) crossed the joint line (%MA)—were measured. These parameters were semi‐automatically measured by one experienced orthopaedic surgeon using planning software (MediCAD; Hectec). This software prompts the examiner to confirm common anatomical landmark points and then automatically measures the alignment parameters described above. As a result, both intra‐observer and inter‐observer errors are minimal, as previously reported [29].

### Arthroscopic observation

All arthroscopic examinations were performed at approximately 1 year postoperatively, coinciding with the surgery to remove the plate and locking screws used for the HTO. Continuity of the repaired portion of the medial meniscus (MM) was assessed using a probing technique. However, it was not possible to objectively evaluate the quality, quantity or function of the tissue generated at the MMPR repair site. Therefore, in this study, the authors avoided subjective descriptions and evaluated only the presence or absence of continuity between the posterior horn and the tibial attachment as an indicator of healing at the MMPR repair site. In addition, cartilage degeneration of the medial femoral condyle was assessed according to the International Cartilage Repair Society (ICRS) classification [14].

### MRI evaluations

For MRI examinations, T2*‐weighted and proton‐depressed images obtained using a 1.5‐Tesla MRI scanner (ECHELON RX™; Hitachi Medico) with an extremity coil were used in this study. In the MRI assessments, first, loss of the MM image at the attachment site was carefully diagnosed. Secondly, the MM extrusion (MME) ratio was defined as the extrusion width (EW) divided by the width of the MM (Figure 2), measured on the coronal MRI slice obtained at the midpoint of the medial tibial plateau, based on the modified Costa's method [5, 32]. Concerning the inter‐rater reliability of the measurements, the width of the MM on the joint line was independently assessed by three orthopaedic surgeons, who were not involved in this study, and the inter‐class coefficients were calculated. To assess intra‐rater reliability, one of the three surgeons repeated the measurements 4 weeks after the initial assessment, and intra‐class correlation coefficients were calculated. Thirdly, the degree of degeneration of the central portion of the MM was assessed using the Mink's classification [23]. In this classification, signal changes within the meniscus were graded as follows: Grade 1, globular increased signal not reaching the articular surface; Grade 2, linear increased signal not reaching the articular surface; Grade 3A, linear abnormal signal extending to the articular surface; and Grade 3B, globular or irregular abnormal signal extending to the articular surface. Grading of degeneration on MRI was independently performed by two experienced knee surgeons. When their assessments differed, they reviewed the MRI findings together and reached a consensus on the final grade.

**Figure 2:**
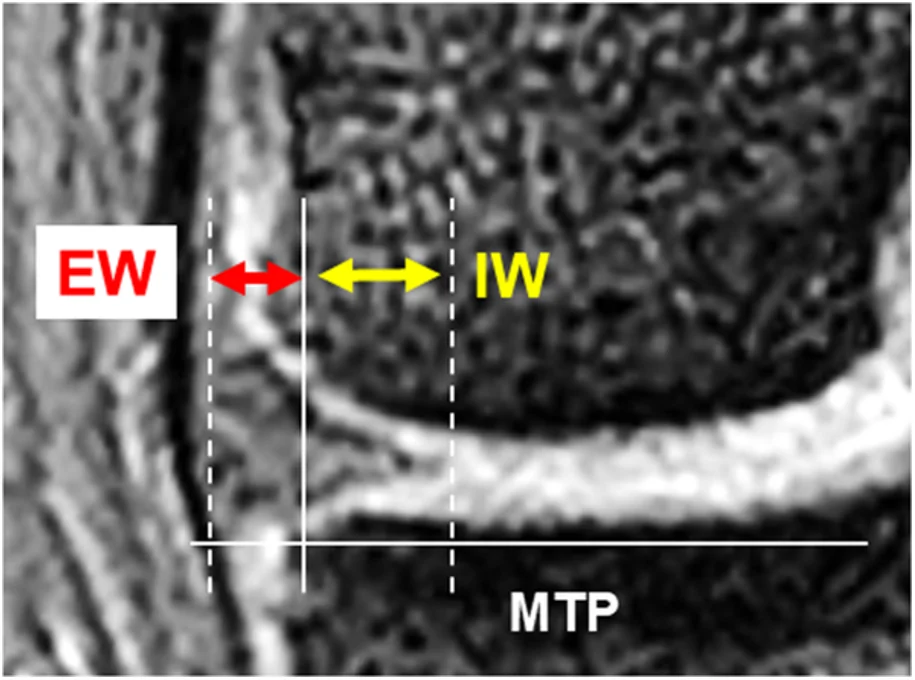
MME was measured on a coronal MRI. The MME ratio was defined as EW/MM width (IW + EW). EW, extrusion width; IW, inner width; MM, medial meniscus; MME, medial meniscus extrusion; MRI, magnetic resonance imaging; MTP, medial tibial plateau.

### Clinical evaluations

Patient demographic data included age, sex, height, body weight, body mass index and radiological OA grade. During the follow‐up examinations, attention was directed toward detecting potential complications, including delayed union at the osteotomy site, infection and osteonecrosis. Specifically, osteonecrosis was monitored by assessing symptoms such as sudden recurrence of knee pain or nocturnal pain, as well as characteristic radiographic findings, and MRI examinations were performed when osteonecrosis was suspected. Any identified complications were documented in the electronic medical record system. In addition, clinical evaluations were performed using the Lysholm score [22] and the Knee Injury and Osteoarthritis Outcome Score (KOOS) [27] at each annual or biennial outpatient visit.

### Statistical analysis

A commercially available software (JMP Pro10.0 for Windows; SAS Institute Japan) was used for statistical analysis. The significance level was set at *p* = 0.05.

The demographic characteristics were compared between the TTP and AIS groups using the Mann–Whitney *U* test for continuous variables and Fisher's exact test for categorical variables. Analyses of each clinical parameter involved comparing preoperative to postoperative changes within each group and comparing the two groups at each time point. To account for multiple comparisons, continuous variables were analysed using linear mixed effects models, binary nominal outcomes using binomial logistic mixed effects models and nominal outcomes with more than two categories using multinomial logistic mixed effects models with a generalized logit link. In all models, group (TTP vs. AIS), time (preoperative vs. postoperative) and the group‑by‑time interaction were included as fixed effects, and knee was included as a random intercept to account for within‑knee correlations arising from repeated measurements. The group‑by‑time interaction was used to assess whether temporal changes differed between the groups. Prespecified model‑based comparisons were conducted to evaluate between‑group differences at each time point. For the MME ratio, a two‑sided exact 95% confidence interval (CI) was calculated using Welch's method to describe the precision of the between‑group difference.

The sample size of this study was determined by the number of eligible knees during the study period because this was a retrospective cohort study. Therefore, an a priori sample size calculation was not feasible. Instead, we emphasize effect estimates with 95% CIs to describe precision. In addition, to assess the sensitivity of our study, we calculated the minimum detectable standardized mean difference based on the available sample size (two‐sided *α* = 0.05, 80% power). With 28 knees in the TTP group and 26 knees in the AIS group, the effect size (Cohen's *d*) was calculated to be 0.76, which indicated smaller effects may not be detectable.

## RESULTS

### Patients

Of the 77 knees (75 patients), all patients underwent a second‐look arthroscopic examination during hardware removal surgery performed 1–2 years after the MMPR repair. The reason for hardware removal in all patients was their preference to avoid retaining substantial foreign material within the body for an extended period. This decision was unrelated to implant‑associated symptoms or infection. Such requests are common in this country, and these preferences may reflect cultural values and health‑related practices, including anticipation of potential future problems. At the minimum 24‐month evaluation, 15 knees (15 patients) were lost to follow‐up because the patients had relocated and were unreachable. In addition, 8 knees (8 patients) were excluded due to the absence of postoperative magnetic resonance imaging (MRI) examinations. Consequently, the remaining 54 knees (52 patients) were included in this study (Figure 3). The TTP group included 28 knees (28 patients), while the AIS group included 26 knees (24 patients). In the TTP group, HTO was performed with the MOW technique in 19 knees and with the iV technique in 9 knees, whereas in the AIS group, it was performed with the MOW technique in 21 knees and with the iV technique in 5 knees. There was no significant difference between the TTP and AIS groups in the proportion of the two HTO procedures performed. The follow‐up period was 51.8 ± 13.0 months (range, 36–70 months) in the TTP group and 33.8 ± 6.1 months (range, 24–48 months) in the AIS group, showing a significant difference between the groups. Therefore, to compare the two groups over an equivalent postoperative follow‐up period, the results of follow‐up evaluations performed 24–48 months postoperatively in the TTP group were used as the evaluation data for that group in this study. All patients in the TTP group had attended at least one outpatient visit during this postoperative period. Among those who had attended two or more outpatient visits, the data were obtained from the follow‑up assessment conducted at the most recent time point within the 24‑ to 48‑month postoperative period. Consequently, the evaluation period in the TTP group was 37.1 ± 4.4 months (range, 24–48 months). Preoperative and follow‐up evaluations included radiographic, arthroscopic, MRI and clinical assessments. Primary comparisons were conducted between the TTP and AIS groups for each measured parameter.

**Figure 3:**
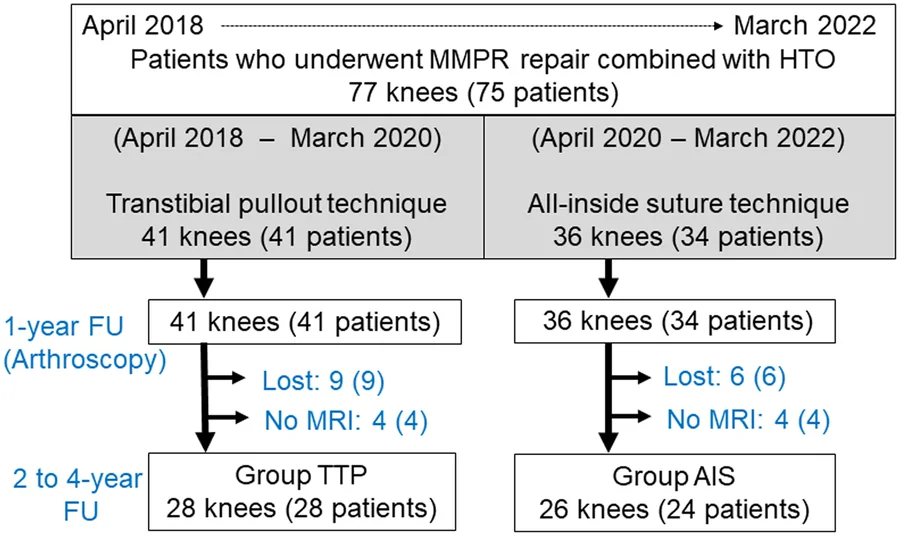
Flowchart of the patients in the two groups. AIS, all‐inside suture technique; HTO, high tibial osteotomy; MMPR, medial meniscus posterior root; MRI, magnetic resonance imaging; TTP, transtibial pullout. All patients underwent a second‐look arthroscopic examination within 1–2 years after surgery.

### Demographic characteristics

Regarding the baseline characteristics of each group, no significant differences were observed between the TTP and AIS groups in age, sex, height, body weight, body mass index (Table 1) or the preoperative distribution of K–L OA grades (Table 2).

**Table 1:** The demographic data of the patients.

	TTP group	AIS group	*p* value
Number of knees (patients)	28 (28)	26 (24)	
Age (years)	61.7 ± 7.6	62.3 ± 8.5	0.709
Male/Female (patients)	4/24	9/17	0.081
Height (cm)	157.2 ± 7.4	157.0 ± 11.4	0.842
Body weight (kg)	63.1 ± 9.9	64.8 ± 14.8	0.795
Body mass index (kg/m^2^)	25.5 ± 3.0	26.0 ± 3.6	0.472

*Note*: The *p* values show the results of comparing each item between the transtibial pullout (TTP) and all‐inside suture (AIS) groups.

**Table 2:** Pre‐ and postoperative distribution of Kellgren–Lawrence OA grades and radiographic alignment parameters in the TTP and AIS groups.

Items evaluated	TTP group	AIS group	*p* value^a^
Knees (patients)	28 (28)	26 (24)	
OA grade (knees, %)			
Preoperative			0.130
Grade 1	10 (35.7%)	3 (11.5%)	
Grade 2	12 (42.9%)	17 (65.4%)	
Grade 3	6 (21.4%)	6 (23.1%)	
Postoperative			0.451
Grade 1	10 (35.7%)	5 (19.2%)	
Grade 2	11 (39.3%)	14 (53.8%)	
Grade 3	7 (25.0%)	7 (26.9%)	
*p* value^b^	0.317	0.655	(0.715)^c^
FTA (°)			
Preoperative	178.2 ± 2.0	178.8 ± 2.5	0.351
Postoperative	170.3 ± 2.1	170.8 ± 1.5	0.453
*p* value^b^	<0.001	<0.001	(0.902)^c^
HKA (°)			
Preoperative	−4.2 ± 2.2	−5.0 ± 2.2	0.134
Postoperative	3.2 ± 1.4	3.0 ± 1.0	0.501
*p* value^b^	<0.001	<0.001	(0.548)^c^
%MA (%)			
Preoperative	28.9 ± 10.8	27.7 ± 9.2	0.641
Postoperative	60.8 ± 8.1	60.2 ± 5.4	0.662
*p* value^b^	<0.001	<0.001	(0.983)^c^
MPTA (°)			
Preoperative	85.0 ± 2.4	85.0 ± 1.7	0.954
Postoperative	92.4 ± 2.2	91.8 ± 2.3	0.324
*p* value^b^	<0.001	<0.001	(0.480)^c^

*Note*: The continuous data are shown as ‘the mean ± the standard deviation’.

Abbreviations: AIS, all‐inside suture; FTA, lateral femorotibial angle; HKA, hip–knee–ankle angle; MA, mechanical axis; MPTA, medial proximal tibial angle; OA, osteoarthritis; TTP, transtibial pullout.

a *p* value: Comparison between the TTP and AIS groups.

b *p* value: Comparison between preoperative and postoperative data within each group.

c (*p* value): Group × time interaction.

### Radiographic evaluations

The postoperative distribution of K–L OA grades did not differ significantly between the groups (Table 2). Within each group, the postoperative distribution of OA grades did not significantly progress compared with the preoperative distribution (Table 2), indicating no significant difference between the two groups. Regarding the preoperative parameters related to lower‐limb alignment, no significant differences in HKA, FTA, %MA or MPTA were found between the TTP and AIS groups (Table 2). The intraoperative correction angle of the tibia was 9.4° ± 2.2 in the TTP group and 8.1° ± 2.2 in the AIS group, with no significant difference (*p* = 0.059). Postoperatively, these parameters showed no significant difference between the TTP and AIS groups (Table 2). Specifically, the postoperative HKA was 3.2° ± 1.4 in the TTP group and 3.0° ± 1.0 in the AIS group, both indicating appropriate valgus alignment for OA knees undergoing HTO.

### Arthroscopic observations

In the preoperative arthroscopy, an MMPR tear was observed in all knees in both groups. At the second‐look arthroscopy performed approximately 1 year postoperatively, discontinuity at the MMPR site was observed in 3 knees (10.7%) in the TTP group and 3 knees (11.5%) in the AIS group, with no significant difference between the groups. In the remaining knees in each group, the posterior root (PR) was reattached to the tibia with fibrous tissue. This fibrous tissue appeared lax, although the degree of laxity could not be quantified. Regarding cartilage degeneration of the medial femoral condyle, the distribution of ICRS grades did not differ significantly between the two groups at either the preoperative or postoperative assessment (Table S1). Within each group, distribution of the ICRS grades did not significantly change between the preoperative and postoperative periods (Table S1).

### MRI evaluations

On preoperative MR images, all knees in both the AIS and TTP groups showed a distinct gap at the MMPR attachment. At the final follow‐up examination, the gap persisted in 10.7% in the TTP group and 11.5% in the AIS group (Table 3). The restoration rate of PR continuity was calculated to be 89.3% in the TTP group and 88.5% in the AIS group, with no significant difference between the groups. The effect of the surgical treatment was significant in each group (*p* < 0.001).

**Table 3:** Pre‐ and postoperative MRI findings of the MM were compared between the TTP and AIS groups.

Items evaluated	TTP group	AIS group	*p* value^a^
Knees (patients)	28 (28)	26 (24)	
Presence of a gap (knees, %)			
Preoperative	28 (100.0%)	26 (100.0%)	1.000
Postoperative	3 (10.7%)	3 (11.5%)	0.923
*p* value^b^	<0.001	<0.001	(0.996)^c^
MME ratio (%)			
Preoperative	49.6 ± 15.3	48.8 ± 16.0	0.579
Postoperative	62.0 ± 18.7	68.8 ± 14.4	0.190
*p* value^b^	0.003	<0.001	(0.064)^c^
Mink's grade of cartilage degeneration (knees, %)			
Preoperative			0.876
0	3 (10.7%)	2 (7.7%)	
1	9 (32.1%)	11 (42.3%)	
2	14 (50.0%)	11 (42.3%)	
3A	2 (7.1%)	2 (7.7%)	
3B	0 (0.0%)	0 (0.0%)	
Postoperative	0 (0.0%)	0 (0.0%)	0.981
0	6 (21.4%)	6 (23.1%)	
1	11 (39.3%)	11 (42.3%)	
2	9 (32.1%)	7 (26.9%)	
3A	2 (7.1%)	2 (7.7%)	
3B			
*p* value^b^	<0.001	<0.001	(0.645)^c^

*Note*: Presence of a gap indicates discontinuity of the MM at the posterior root. MME indicates medial meniscus extrusion. Cartilage degeneration in the medial meniscus mid‐substance was assessed with the Mink's grade.

Abbreviations: AIS, all‐inside suture; MM, medial meniscus; MME, medial meniscus extrusion; MRI, magnetic resonance imaging; TTP, transtibial pullout.

a *p* value: Comparison between the TTP and AIS groups.

b *p* value: Comparison between preoperative and postoperative data within each group.

c (*p* value): Group × time interaction.

Regarding the MME ratio, the intra‐class coefficients were 0.950 (95% CI: 0.896–0.979) and 0.994 (0.987–0.997), respectively. The inter‐class coefficients were 0.865 (0.740–0.939) and 0.980 (0.960–0.990), respectively. These findings indicate that both intra‐ and inter‐rater reliability for the measurement of the MME ratio were satisfactory. The preoperative mean MME ratio was 49.6% (95% CI: 43.7−55.5) in the TTP group and 48.8% (42.3−55.3) in the AIS group, with no significant difference between the two groups (Table 3). At the final follow‐up, the mean ratio significantly increased to 62.0% (54.7−69.3) in the TTP group (*p* = 0.003) and to 68.8% (63.0–74.6) in the AIS group (*p* < 0.001), while there was no significant difference between the two groups (mean difference [TTP − AIS], 6.8%) (Table 3). However, the increase was larger in the AIS group (average, 20.0%) than in the TTP group (12.4%). In addition, the power analysis indicated only 76% power to detect a large effect. Accordingly, the existence of intergroup differences in the progression of MME cannot be definitively excluded. The proportions of knees showing an increase of ≥10%, no change (−10% to 10%) or a decrease of ≤−10% in the MME ratio were 57.1%, 32.1% and 10.7% in the TTP group, and 73.1%, 23.1% and 3.8% in the AIS group, respectively, with no significant differences between the groups.

Regarding degeneration of the MM mid‐substance (Table 3), each group exhibited a significant shift in the distribution of Mink grades from the preoperative to the postoperative period (*p* < 0.001). The distribution did not significantly differ between the two groups at either the preoperative or postoperative assessment (Table 3).

### Clinical evaluations

Preoperatively, there were no significant differences in Lysholm and KOOS subscale scores between the two groups (Table 4). Postoperatively, no complications, such as infection, osteonecrosis or delayed union at the osteotomy site, occurred in either group. The postoperative Lysholm score improved significantly to 96.4 and 92.8 points, respectively, and all KOOS subscales also showed significant improvement compared with preoperative values (*p* < 0.001), with no significant differences observed between the two groups (Table 4). The proportions of patients achieving the minimal clinically important difference (MCID) and the patient acceptable symptom state (PASS) were comparable between the two groups, with no statistically significant differences (Tables S2 and S3).

**Table 4:** Pre‐ and postoperative Lysholm score and KOOS in the TTP and AIS groups.

Items evaluated	TTP group	AIS group	*p* value^a^
Knees (patients)	28 (28)	26 (24)	
Lysholm score (points)			
Preoperative	64.5 ± 12.9	58.8 ± 12.8	0.138
Postoperative	96.3 ± 4.5	92.8 ± 8.2	0.083
*p* value^b^	<0.001	<0.001	(0.570)^c^
*KOOS (points)*			
(Pain)			
Preoperative	53.1 ± 14.8	55.8 ± 21.2	0.290
Postoperative	86.6 ± 15.1	93.2 ± 6.1	0.567
*p* value^b^	<0.001	<0.001	(0.788)^c^
(Symptoms)			
Preoperative	58.7 ± 14.8	65.1 ± 16.5	0.138
Postoperative	86.6 ± 10.3	90.4 ± 6.6	0.108
*p* value^b^	<0.001	<0.001	(0.957)^c^
(ADL)			
Preoperative	67.8 ± 14.7	67.1 ± 13.4	0.855
Postoperative	87.2 ± 15.3	93.6 ± 5.3	0.173
*p* value^b^	<0.001	<0.001	(0.286)^c^
(Sports/Rec.)			
Preoperative	25.6 ± 24.1	32.5 ± 18.7	0.605
Postoperative	68.2 ± 16.1	76.6 ± 16.4	0.124
*p* value^b^	<0.001	<0.001	(0.554)^c^
(QOL)			
Preoperative	26.2 ± 12.1	28.4 ± 19.9	0.653
Postoperative	71.5 ± 16.2	77.3 ± 14.3	0.236
*p* value^b^	<0.001	<0.001	(0.645)^c^

*Note*: The data are shown as ‘the mean ± the standard deviation’.

Abbreviations: ADL, activities of daily living; AIS, all‐inside suture; KOOS, Knee injury and Osteoarthritis Outcome Score; QOL, quality of life; Sports/Rec, sports/recreation; TTP, transtibial pullout.

a *p* value: Comparison between the TTP and AIS groups.

b *p* value: Comparison between preoperative and postoperative data within each group.

c (*p* value): Group × time interaction.

## DISCUSSION

The main findings of the present study were that, among patients undergoing MMPR repair combined with HTO, no significant differences were observed between the TTP and AIS groups in demographic characteristics, radiographic parameters, arthroscopic findings, MRI assessments or clinical outcomes at 24–48 months of follow‐up. Specifically, the distribution of K–L grades did not show significant postoperative progression in either group. Both groups demonstrated comparable rates of PR continuity restoration (89.3% vs. 88.5%). The Lysholm score improved to 96.3 ± 4.5 and 92.8 ± 8.1 points significantly, respectively (*p* < 0.001), and all KOOS subscales also showed significant improvement compared with preoperative values. Notably, however, the degree of MME and the Mink grade of degeneration in the MM mid‐substance increased significantly at follow‐up in both cohorts.

Only a few studies have compared the relative superiority of the TTP and AIS techniques in MMPR repair performed with HTO. Lee et al. [20] found no significant differences in clinical outcomes between the two techniques in patients with severe knee OA, whereas Omae et al. [27] reported better healing rates and clinical outcomes with the AIS technique. In the present study, radiological parameters and clinical outcomes were comparable between the TTP and AIS techniques. Because HTO reduces the load on the medial compartment, differences in the relative superiority of the techniques may be difficult to detect. Thus, our findings suggest that comparable outcomes can be expected regardless of which technique is used when MMPR repair is performed with HTO.

Previous studies of MMPR repair without concomitant HTO have reported favourable healing rates at the suture site; for example, Chang et al. [2] found ‘complete or partial healing’ in 94% of cases. However, these studies lacked objective criteria for evaluating healing and did not assess the functional status of the repaired meniscus. In the present study, PR continuity was restored in most cases in both the TTP and AIS groups, yet the postoperative MME ratio increased markedly (from 49.6% to 62.0% in the TTP group and from 48.8% to 68.8% in the AIS group), indicating reduced meniscal function. Thus, apparent continuity at the repair site may not reflect true functional recovery. Because many previous studies did not measure MME, future research should include MME assessment on MRI as an indicator of repaired meniscal function.

Among previous studies of MMPR repair combined with HTO, only two reported postoperative MME ratios [15, 25]. Ke et al. [15] found no significant change in MME, even in knees with extensive resection of the torn MMPR portion. In contrast, Omae et al. [25] observed a postoperative decrease in MME in knees with complete healing, but no change in those with poor healing. These findings differ from those of the present study. Conversely, most studies of MMPR repair without concomitant HTO have reported increased postoperative MME. Moon et al. [24] and Kaplan et al. [14] both noted postoperative increases, and a systematic review by Chang et al. [2] found similar results in five of nine studies. More recently, Yoon et al. [34] also reported increased MME after both TTP and AIS procedures. Thus, the present findings align with the majority of reports on MMPR repair performed without concomitant HTO.

A previous review of MMPR repair without concomitant HTO reported radiographic progression of at least one K–L grade in about half of patients [2]. More recently, Dzidzishvili et al. [9] showed that knees with >5° varus alignment had greater OA progression after MMPR repair than those with <5°, emphasizing the importance of baseline alignment for postoperative joint survival. In contrast, in the present study of MMPR repair combined with HTO, no significant K–L grade progression or cases of medial femoral condyle osteonecrosis were observed at 2–4 years, consistent with the findings of Lee et al. [20]. These results suggest that adding HTO may suppress OA progression and osteonecrosis despite increased MME and may also contribute to improved clinical outcomes. Recently, Cortina et al. [6] reviewed eight retrospective studies and reported that MMPR repair combined with HTO yields statistically better short‐term IKDC scores. However, they also noted that the long‐term benefit of this combined procedure remains uncertain. Thus, longer‐term studies are needed to clarify the effects of isolated MMPR repair and the additional impact of HTO in knees with mild OA and an MMPR tear.

A major limitation of this study is its retrospective sequential cohort design, in which treatment‐related background factors may differ between study periods. These factors include patient selection, baseline characteristics, imaging protocols, surgeon experience, indications for HTO and rehabilitation and follow‐up duration. In the present study, however, the inclusion criteria remained consistent, and no significant intergroup differences were found in demographic variables, preoperative OA grades, the MOWHTO/iVHTO ratio or postoperative alignment. Imaging protocols and equipment were unchanged. Regarding potential learning curve effects, the TTP technique had been used for more than one year before the study commenced. The rationale for subsequently switching to the AIS technique was that the AIS technique is technically less complex and less demanding than the TTP technique [7] and is associated with a shorter operative duration. In addition, the AIS technique using the FAST FIX 360™ device was identical to that employed in numerous prior cases of radial meniscal tears in the authors' clinical practice. Therefore, learning curve effects were considered unlikely to have influenced the outcomes. The indications for HTO and the rehabilitation protocol were also consistent. To address the difference in follow‐up duration between the two groups, only the TTP data obtained at postoperative time points corresponding to those of the AIS group were used, thereby aligning the observation periods between the two groups. However, the possibility of secular trends not only in the factors evaluated in this study but also in other unevaluated factors cannot be completely excluded. Therefore, future Level I studies are needed to confirm these findings, although the present results provide important preliminary information for designing such high‐quality research.

The second limitation of this study is that two types of HTO procedures were used depending on the required correction angle. Previous studies have shown that postoperative alignment, rather than the osteotomy technique, determines the therapeutic effect of HTO [1, 19, 33], and comparable outcomes have been reported for MOWHTO and iVHTO when alignment is equivalent [10, 19]. In the present study, postoperative alignment did not differ between the TTP and AIS groups. In addition, a supplementary analysis comparing demographic characteristics, radiological parameters and clinical outcomes between MOWHTO and iVHTO revealed no significant differences (Tables [Link], [Link], [Link], [Link]). However, the statistical power of this analysis was limited (56%–86%). Thus, the possibility that differences between the two HTO procedures influenced the outcomes cannot be definitively excluded.

There are several additional limitations to this study. First, the number of cases included was not sufficiently large to allow definitive conclusions to be drawn. Second, the follow‐up duration differed between the two groups, which may have influenced the comparability of the outcomes. Third, long‐term follow‐up has not yet been achieved for the included cases, leaving potential long‐term differences in clinical outcomes between the two groups unclear. Fourth, this study did not include any cases in which HTO was performed alone without concomitant MMPR repair. Therefore, the therapeutic effects demonstrated for MMPR repair combined with HTO cannot be directly compared with those of isolated HTO. Despite these limitations, the present study provides valuable new insights into the surgical management of MMPR tears.

In conclusion, the two groups had comparable demographic characteristics. Radiologically, K–L grades showed no postoperative progression or intergroup differences. MRI and arthroscopy demonstrated similar PR continuity rates (89.3% in TTP and 88.5% in AIS). The mean MME ratio increased significantly in both groups without intergroup differences. The Lysholm score and all KOOS subscales improved significantly in both groups with no significant differences. Overall, in this patient population, the two repair techniques combined with HTO yielded comparable radiological and clinical improvements at 24–48 months of follow‐up, despite increased MME.

## AUTHOR CONTRIBUTIONS

All authors have reviewed and approved the final version of the manuscript. Yuki Fujie performed data collection, data analysis, data interpretation and drafted the manuscript. Kazunori Yasuda and Eiji Kondo developed the initial concept, provided the data, performed data interpretation and critically appraised the manuscript. Koji Yabuuchi and Taku Ebata performed data analysis, data interpretation, statistical analysis and critically appraised the manuscript. Masayuki Inoue and Norimasa Iwasaki performed data analysis and critically appraised the manuscript.

## FUNDING INFORMATION

The authors have no funding to report.

## CONFLICT OF INTEREST STATEMENT

The authors declare no conflicts of interest.

## ETHICS STATEMENT

The study was approved by the local ethical committee (Yagi Orthopaedic Hospital, Sapporo, Japan, 2017‐04‐8) and the clinical trial was conducted in agreement with the 1964 Declaration of Helsinki and subsequent amendments. All patients signed a consent statement for the use of their data for scientific purposes.

## Supporting information

Supporting File 1.

Supporting File 2.

Supporting File 3.

Supporting File 4.

Supporting File 5.

Supporting File 6.

Supporting File 7.

Supporting File 8.

## Data Availability

The data that support the findings of this study are available in the supporting information of this article.
