# Supplementary material for: Posterior root repair with high tibial osteotomy improves radiological and clinical outcomes despite greater medial meniscus extrusion: A comparison of transtibial and all‐inside techniques
Source: J Exp Orthop. 2026 Oct 19;13(4):e70956. doi: 10.1002/jeo2.70956 (PMC13680541; doi:10.1002/jeo2.70956)
Supplement: Supplementary file 1 [file JEO2-13-e70956-s001.docx]

**Supplementary Table 5.** Comparisons of the pre- and postoperative Kellgren–Lawrence osteoarthritis grades of patients between the medial opening wedge (MOW) and inverted V-shaped (iV) HTO groups in the present study. No significant differences were observed between the groups.

| Items evaluated | OWHTO group | iVHTO group | *P value* |
| --- | --- | --- | --- |
| Number of knees (patients) | 40 | 14 |  |
| OA grade (knees, %) |  |  |  |
| Preoperative Grade 1 | 9 (22.5) | 4 (28.6) | 0.899 |
| Grade 2 | 22 (55.0) | 7 (50.0) |  |
| Grade 3 | 9 (22.5) | 3 (21.4) |  |
| Postoperative Grade 1 | 11 (27.5) | 4 (28.6) | 0.950 |
| Grade 2 | 19 (47.5) | 6 (42.9) |  |
| Grade 3 | 10 (25.0) | 4 (28.6) |  |
