# Supplementary material for: Posterior root repair with high tibial osteotomy improves radiological and clinical outcomes despite greater medial meniscus extrusion: A comparison of transtibial and all‐inside techniques
Source: J Exp Orthop. 2026 Oct 19;13(4):e70956. doi: 10.1002/jeo2.70956 (PMC13680541; doi:10.1002/jeo2.70956)
Supplement: Supplementary file 2 [file JEO2-13-e70956-s002.docx]

**Supplementary Table 2.** Proportion of patients achieving the minimal clinically important difference (MCID) in the Lysholm score and the Knee Injury and Osteoarthritis Outcome Score (KOOS). Data were reported as number of patients achieving MCID / total number of patients (%).

|  | MCID threshold (points) | Group TTP | Group AIS | P value |
| --- | --- | --- | --- | --- |
| Lysholm score (points) | 8.9 | 26/28 (92.9%) | 25/26 (96.2%) | *P = 1.000* |
| KOOS (points) |  |  |  |  |
| (Pain) | 15.4 | 26/28 (92.9%) | 21/26 (80.8%) | *P = 0.243* |
| (Symptom) | 15.1 | 20/28 (71.4%) | 18/26 (69.2%) | *P = 1.000* |
| (ADL) | 17.0 | 20/28 (71.4%) | 21/26 (80.8%) | *P = 0.530* |
| (Sport / Rec) | 11.2 | 22/28 (78.6%) | 22/26 (84.6%) | *P = 0.730* |
| (QOL) | 16.5 | 24/28 (85.7%) | 24/26 (92.3%) | *P = 0.670* |
