# Supplementary material for: Posterior root repair with high tibial osteotomy improves radiological and clinical outcomes despite greater medial meniscus extrusion: A comparison of transtibial and all‐inside techniques
Source: J Exp Orthop. 2026 Oct 19;13(4):e70956. doi: 10.1002/jeo2.70956 (PMC13680541; doi:10.1002/jeo2.70956)
Supplement: Supplementary file 3 [file JEO2-13-e70956-s003.docx]

**Supplementary Table 7.** Comparisons of the pre- and postoperative Lysholm scores and Knee injury and Osteoarthritis Outcome Score (KOOS) subscales of patients between the medial opening wedge (MOW) and inverted V-shaped (iV) HTO groups in the present study. No significant differences were observed in the scores between the groups. The data are presented as mean ± standard deviation.

| Items evaluated | OWHTO group | iVHTO group | *P value* |
| --- | --- | --- | --- |
| Number of knees (patients) | 40 (37) | 14 (14) |  |
| Preoperative |  |  |  |
| Lysholm score (points) | 61.9 ± 14.1 | 61.4 ± 9.7 | 0.797 |
| KOOS *(Pain)* | 53.1 ± 18.0 | 60.8 ± 21.6 | 0.357 |
| *(Symptoms)* | 59.7 ± 16.0 | 68.1 ± 10.6 | 0.081 |
| *(Activity of daily living)* | 65.3 ± 13.4 | 75.0 ± 12.6 | 0.089 |
| *(Sports / Recreation)* | 26.5 ± 18.9 | 42.2 ± 24.4 | 0.073 |
| *(Quality of life)* | 26.0 ± 16.9 | 34.9 ± 16.1 | 0.072 |
| Postoperative |  |  |  |
| Lysholm score (points) | 94.4 ± 7.3 | 95.2 ± 5.0 | 0.974 |
| KOOS *(Pain)* | 91.4 ± 7.0 | 85.5 ± 20.1 | 0.950 |
| *(Symptoms)* | 89.6 ± 7.5 | 85.3 ± 11.5 | 0.234 |
| *(Activity of daily living)* | 92.0 ± 7.6 | 85.7 ± 19.1 | 0.346 |
| *(Sports / Recreation)* | 71.3 ± 20.8 | 66.9 ± 18.8 | 0.302 |
| *(Quality of life)* | 75.7 ± 15.4 | 70.5 ± 15.4 | 0.435 |
