# Supplementary material for: Posterior root repair with high tibial osteotomy improves radiological and clinical outcomes despite greater medial meniscus extrusion: A comparison of transtibial and all‐inside techniques
Source: J Exp Orthop. 2026 Oct 19;13(4):e70956. doi: 10.1002/jeo2.70956 (PMC13680541; doi:10.1002/jeo2.70956)
Supplement: Supplementary file 5 [file JEO2-13-e70956-s005.docx]

**Supplementary Table 3.** Proportion of patients achieving the patient acceptable symptom state (PASS) in the Knee Injury and Osteoarthritis Outcome Score (KOOS). Data were reported as number of patients achieving PASS / total number of patients (%).

|  | PASS threshold (points) | Group TTP | Group AIS | P value |
| --- | --- | --- | --- | --- |
| KOOS (points) |  |  |  |  |
| (Pain) | 72.2 | 27/28 (96.4%) | 26/26 (100.0%) | *P = 1.000* |
| (Symptom) | 71.4 | 26/28 (92.9%) | 25/26 (96.2%) | *P = 1.000* |
| (ADL) | 77.9 | 25/28 (89.3%) | 26/26 (100.0%) | *P = 0.237* |
| (Sport / Rec) | 40.0 | 27/28 (96.4%) | 25/26 (96.2%) | *P = 1.000* |
| (QOL) | 56.3 | 20/28 (71.4%) | 22/26 (84.6%) | *P = 0.332* |
