# Supplementary material for: Posterior root repair with high tibial osteotomy improves radiological and clinical outcomes despite greater medial meniscus extrusion: A comparison of transtibial and all‐inside techniques
Source: J Exp Orthop. 2026 Oct 19;13(4):e70956. doi: 10.1002/jeo2.70956 (PMC13680541; doi:10.1002/jeo2.70956)
Supplement: Supplementary file 6 [file JEO2-13-e70956-s006.docx]

**Supplementary Table 6.** Comparisons of the pre- and postoperative MRI evaluations of patients between the medial opening wedge (MOW) and inverted V-shaped (iV) HTO groups in the present study. No significant differences were observed in the gap (discontinuity) at the MMPR and the medial meniscus extrusion (MME) ratio between the groups.

| Items evaluated | OWHTO group | iVHTO group | *P value* |
| --- | --- | --- | --- |
| Number of knees (patients) | 40 (37) | 14 (14) |  |
| Presence of a Gap (knees, %) |  |  |  |
| Preoperative | 40 (100) | 14 (100) | *1.000* |
| Postoperative | 7 (17.5) | 2 (14.3) | *1.000* |
| MME ratio (%) |  |  |  |
| Preoperative | 48.2 ± 13.8 | 52.1 ± 19.8 | *0.353* |
| Postoperative | 65.3 ± 16.8 | 65.0 ± 18.0 | *0.836* |
