# Supplementary material for: Posterior root repair with high tibial osteotomy improves radiological and clinical outcomes despite greater medial meniscus extrusion: A comparison of transtibial and all‐inside techniques
Source: J Exp Orthop. 2026 Oct 19;13(4):e70956. doi: 10.1002/jeo2.70956 (PMC13680541; doi:10.1002/jeo2.70956)
Supplement: Supplementary file 7 [file JEO2-13-e70956-s007.docx]

**Supplementary Table 1.** Pre‑ and postoperative arthroscopic observations of cartilage degeneration (ICRS grading) on the medial femoral condyle in the transtibial pullout (TTP) and all‑inside suture (AIS) groups.

| Items evaluated | TTP group | AIS group | *P value ^a^* |
| --- | --- | --- | --- |
| Number of knees (patients) | 28 (28) | 26 (24) |  |
| *Preoperative* Grade 1  Grade 2  Grade 3  Grade 4 | 4 (14.3%)  10 (35.7%)  9 (32.1%)  5 (17.9%) | 7 (26.9%)  4 (15.4%)  8 (30.8%)  7 (26.9%) | *0.294* |
| *Postoperative* Grade 1  Grade 2  Grade 3  Grade 4 | 6 (21.4%)  9 (32.1%)  7 (25.0%)  6 (21.4%) | 6 (23.0%)  9 (34.6%)  5 (19.2%)  6 (23.1%) | *0.853* |
| *P value ^b^* | *0.256* | *0.723* | *(0.991) ^c^* |

P value ᵃ: Comparison between the TTP and AIS groups.

P value ᵇ: Comparison between preoperative and postoperative data within each group.

(P value) ᶜ: Group × time interaction.
