# Supplementary material for: Posterior root repair with high tibial osteotomy improves radiological and clinical outcomes despite greater medial meniscus extrusion: A comparison of transtibial and all‐inside techniques
Source: J Exp Orthop. 2026 Oct 19;13(4):e70956. doi: 10.1002/jeo2.70956 (PMC13680541; doi:10.1002/jeo2.70956)
Supplement: Supplementary file 8 [file JEO2-13-e70956-s008.docx]

**Supplementary Table 4.** Comparisons of the demographic characteristics of patients between the medial opening wedge (MOW) and inverted V-shaped (iV) HTO groups in the present study. No significant differences were observed between the groups. Abbreviations: TTP, transtibial pullout; AIS, all-inside suture.

| Items | OWHTO group | iVHTO group | P value |
| --- | --- | --- | --- |
| Number of knees (patients) | 40 (37) | 14 (14) |  |
| Age (years) | 61.7 ± 8.9 | 62.9 ± 6.0 | 0.961 |
| Male / Female (patients) | 9 / 31 | 4 / 10 | 0.722 |
| Height (cm) | 156.9 ± 9.3 | 157.6 ± 10.9 | 0.961 |
| Body weight (kg) | 64.2 ± 12.7 | 63.4 ± 12.9 | 0.782 |
| Body mass index (kg/m²) | 25.9 ± 3.5 | 25.3 ± 3.1 | 0.782 |
| Repair procedure TTP | 19 (47.5) | 9 (64.3) | 0.358 |
| (knees, %) AIS | 21 (52.5) | 5 (35.7) |  |
